# Non-isothermal thermogravimetric kinetic analysis of the thermochemical conversion of human faeces

**DOI:** 10.1016/j.renene.2018.08.090

**Published:** 2019-03

**Authors:** B. Fidalgo, M. Chilmeran, T. Somorin, A. Sowale, A. Kolios, A. Parker, L. Williams, M. Collins, E.J. McAdam, S. Tyrrel

**Affiliations:** School of Water, Energy and Environment, Cranfield University, MK43 0AL, UK

**Keywords:** Pyrolysis, Combustion, Thermogravimetric analysis, Kinetics, Human faeces, Nano membrane toilet

## Abstract

The “Reinvent the Toilet Challenge” set by the Bill & Melinda Gates Foundation aims to bring access to adequate sanitary systems to billions of people. In response to this challenge, on-site sanitation systems are proposed and being developed globally. These systems require in-situ thermal treatment, processes that are not well understood for human faeces (HF). Thermogravimetric analysis has been used to investigate the pyrolysis, gasification and combustion of HF. The results are compared to the thermal behaviour of simulant faeces (SF) and woody biomass (WB), along with the blends of HF and WB. Kinetic analysis was conducted using non-isothermal kinetics model-free methods, and the thermogravimetric data obtained for the combustion of HF, SS and WB. The results show that the devolatilisation of HF requires higher temperatures and rates are slower those of WB. Minimum temperatures of 475 K are required for fuel ignition. HF and SF showed similar thermal behaviour under pyrolysis, but not under combustion conditions. The activation energy for HF is 157.4 kJ/mol, relatively higher than SS and WB. Reaction order for HF is lower (n = 0.4) to WB (n = 0.6). In-situ treatment of HF in on-site sanitary systems can be designed for slow progressive burn.

## Highlights

• Human faeces (HF) require minimum temperatures of 475 K for devolatilisation.
• Maximum conversion rates for combustion of HF occurred at about 710 and 880 K.
• Woody biomass improves combustion of HF; higher conversion rates achieved.
• Human faeces and synthetic faeces have similar thermal behaviour under pyrolysis.
• Average activation energy for human faeces is 157.4 kJ/mol; can be up to 450 kJ/mol.

## 1 Introduction

The Joint Monitoring Programme (JMP) for Water Supply, Sanitation and Hygiene reported that 32% of the global population had no access to basic sanitation services in 2015 [1]. In many developing countries, more than 90% of the faeces generated are not safely disposed, which poses serious health and environmental threats [1]. The “Reinvent the Toilet Challenge” set by Bill & Melinda Gates Foundation aims at the development and deployment of novel sanitary systems without connections to water, sewer and electrical supplies in order to ensure safe, affordable and sustainable sanitation solutions to people worldwide [2]. New concepts of sanitary systems have arisen as response to this challenge. The Nano Membrane Toilet (NMT), which is being developed at Cranfield University, is based on the recognition of human faeces (HF) as a fuel instead of a waste. The NMT includes the in-situ combustion of HF to produce energy for self-sustained operation of the unit [3]. HF is a carbon-based fuel which consists of a mixture of undigested fat, protein, water, polysaccharide, bacterial biomass, gut secretions, cell shedding and ash [4], and can be converted into energy via gasification and combustion [3,5,6].

In order to efficiently use human faeces as feedstock of combustion units and achieve the advantages that it can bring as a fuel, more information and understanding of the thermochemical characteristics of this material is required. Thermogravimetric analysis (TGA) has been largely employed to investigate the thermal process of coal and biomass fuels, including animal manure and municipal and industrial sludges [[7], [8], [9], [10], [11], [12], [13], [14], [15], [16], [17], [18]], with the objective of assessing the materials as fuel, establishing optimum operating conditions and investigating the kinetics of the process. Most of the published works focus on the co-processing of sludge with coal or biomass, exhibiting that co-combustion of these fuels can be viable from the energetic, economic and environmental point of view. Fewer examples of the thermal behaviour of individual sludge and manure fuels can be found in literature, and most of them are focused on pyrolysis [[19], [20], [21], [22], [23], [24], [25]]. Sanchez et al. [23] investigated the combustion of sewage sludge and animal manure by thermogravimetric analysis, and applied the Ozawa-Flynn-Wall (OFW) model to calculate the activation energy of the process. They concluded that the combustion of these biowastes is complex, changes with the degree of conversion, and is characteristic of each material; however, they found similar average activation energy (∼140 kJ/mol) and average reaction order (∼0.15) for the combustion of both the sludge and the manure. Abbas et al. [24] studied the combustion of dewatered sewage sludge and the kinetics of the process by means of OFW and the Vyazovkin models. They established average activation energy values for combustion (82 and 79 kJ/mol depending on the applied model). The authors also observed that the combustion reaction order varied with conversion and determined an average value of 0.11. Wu et al. [25] reported the combustion of dairy manure to occur in four stages, namely drying, first oxidation zone including the highest weight loss, second oxidation zone, and third oxidation zone related to the combustion of the char residue. The authors applied a kinetic model based on the Arrhenius equation and determined an activation energy value of 83 kJ/mol and reaction order of 5.24 for the first oxidation stage, and values of 56 kJ/mol and 1.25 for the second oxidation stage.

Pyrolysis, gasification and combustion behaviour of human faeces, and associated kinetic studies, have not been reported in literature. As previous work of our research group, Onabanjo et al. [5] carried out an experimental investigation of the combustion of human faeces in a bench-scale downdraft reactor and achieved fuel burn rates of 1.5–2.3 g/min at air flow rates of 10–18 L/min. Kinetic results obtained from sewage sludge and simulant faeces could be useful as initial approach to design systems for human faeces combustion; however, most of the available data refer to the thermal behaviour of blends of these materials with coal or biomass. Further research on the thermochemical behaviour of human faeces is essential in order to design the next-generation of non-sewered sanitary systems with in-situ thermal treatment, such as the NMT.

The aim of this work is to investigate the thermochemical conversion of human faeces by means of thermogravimetric analysis. Three different reaction atmospheres, i.e. N_2_, CO_2_, and air, were used to study thermal conversion under pyrolysis, gasification and combustion conditions. Samples of simulant faeces, woody biomass, and blends of human faeces and woody biomass were also evaluated for comparison purposes. Non-isothermal kinetics model-free methods are applied for the first time to assess the kinetics of combustion of human faeces. The research provides new data for understanding the thermochemical conversion of human faeces, which is fundamental to design onsite sanitary energy technologies, such as the NMT.

## 2 Material and methods

Human faeces were collected and stored in a freezer at 188 K (−85 °C) to prevent microbial degradation. The human faeces were collected, stored and manipulated under the approved procedures of the Cranfield University Research Ethics Scheme. Previous to testing, the frozen samples were thawed at room temperature and mixed until a uniform consistency was obtained. The homogenised human faeces sample was dried to a constant weight at 45 °C in a GENLAB Hot Air Oven. The thermal behaviour of HF was compared with those of simulant faeces (SF) and woody biomass (WB). SF was prepared using the recipe described elsewhere [5]. WB was sourced locally. In addition, the thermal behaviour of blends of the HF and WB samples were also analysed; the mixtures consisted of 50, 60, 70 and 80 wt% of HF.

The thermal behaviour of the samples was evaluated through a series of experiments carried out in a Perkin Elmer “Pyris 1” thermogravimetric (TG) analyser in duplicates. Thermal conversion was tested under three reaction atmospheres, i.e. N_2_ for pyrolysis, CO_2_ for gasification and air for combustion conditions. All tests were performed on 20 ± 0.5 mg of sample and 45 mL/min of gas flow rate. The temperature was first set at 323 K for 1 min for weight stabilisation, and then constantly heated to a temperature of 1223 K. The sample was kept at the 1223 K for 2 min to ensure complete conversion. In the case of experiments performed with single samples, three heating rates were tested, namely 5, 25 and 50 K/min. All tests with HF and WB blends were performed at a heating rate of 50 K/min.

The mass ratio of the samples is expressed as given by equation (1).

(1) $$R=\;\frac{m_{t}}{m_{0}}$$

where R is the mass ratio, m_0_ is the initial mass of the sample, and m_t_ is the time dependent mass.

HF, SF and WB samples presented different content of ash. The ash content of WB was virtually negligible (0.6 wt%), while those of SF (13.5 wt%) and HF (19.7 wt%) were high. For comparative purpose, the mass ratio of the samples was calculated in dry ash free (daf) basis as shown in equation (2).

(2) $$R'=\;\frac{m_{t}-\;m_{ash}}{m_{0}-\;m_{ash}}$$

where R′ is the mass ratio in daf basis and m_ash_ is the mass of ash established as the sample mass at the end of the combustion process.

Tests performed with single samples were labelled as X-Y-Z, where X refers to the type of sample (i.e. X = HF, SF or WB), Y refers to the reaction atmosphere (i.e. Y = N2, CO2 or air), and Z refers to the heating rate (i.e. Z = 5, 25 or 50 K/min). The analyses with blends were labelled as xHF-Y-50, where x refers to the percentage of HF in the mixture (i.e. x = 50, 60, 70 or 80) and, as above, Y refers to the reaction atmosphere (i.e. Y = N2, CO2 or air), and 50 refers to a heating rate of 50 K/min.

### 2.1 Determination of combustion kinetics

Non-isothermal kinetics were investigated through model-free methods, which are flexible to allow for changes in mechanism during the course of the reaction [23,27]. The rate of thermal decomposition gas-solid reactions can be described by equation (3) [23].

(3) $$\frac{d\alpha }{dt}=k\left(T\right)f\left(\alpha \right)$$

(4) $$\alpha =\;\frac{m_{0}\;-\;\;m_{t}}{m_{0}\;-\;m_{f}}\;$$

where α is the degree of conversion at any time calculated by equation (4), t is time, T is temperature, k(T) is the temperature-dependent kinetic constant, and f(α) is the derivative representation of the reaction model. In equation (4), m_f_ is the final mass of the sample.

The temperature dependence of the kinetic constant is described by the Arrhenius equation and the heating rate (β = dT/dt) can be inserted in equation (4) resulting into equation (5). This expression describes the rate of combustion reaction as a function of temperature at a constant heating rate.

(5) $$\frac{d\alpha }{dT}=\frac{1}{\beta }Ae^{-E_{a}/RT}f\left(\alpha \right)$$

where β is the heating rate, E_a_ is the activation energy in kJ/mol and A is the pre-exponential factor.

Activation energy values from the experimental data were calculated from two model-free methods, the Ozawa-Flynn-Wall (OFW, equation (6)) and the Vyazovkin (Vy, equation (7)) approaches [23,[27], [28], [29], [30]]. Both methods require the determination of temperature at fixed values of α from tests at different heating rates. OFW and Vy methods allow the investigation of the thermochemical conversion kinetics and activation energy without determining the reaction order.

(6) $$\log\;\beta =\log\frac{AE_{a}}{Rg\left(\alpha \right)}-2.315-0.457\frac{E_{a}}{RT}$$

(7) $$\ln\frac{\beta }{T^{2}}=\ln\frac{RA}{E_{a}g\left(\alpha \right)}-\frac{E_{a}}{R}\frac{1}{T}$$

where g(α) is the integral representation of the reaction model.

Avrami's theory [23,24] (equation (8)) was applied to calculate the value of the reaction order, *n*, at different temperatures by determining the values of α at fixed temperatures from tests at different heating rates.

(8) $$\ln\left[-\ln\left(1-\text{α}\right)\right]=lnA-\frac{E_{a}}{RT}-n\;\ln\;\beta$$

## 3 Results and discussion

### 3.1 TGA results for human faeces

**Fig. 1:**
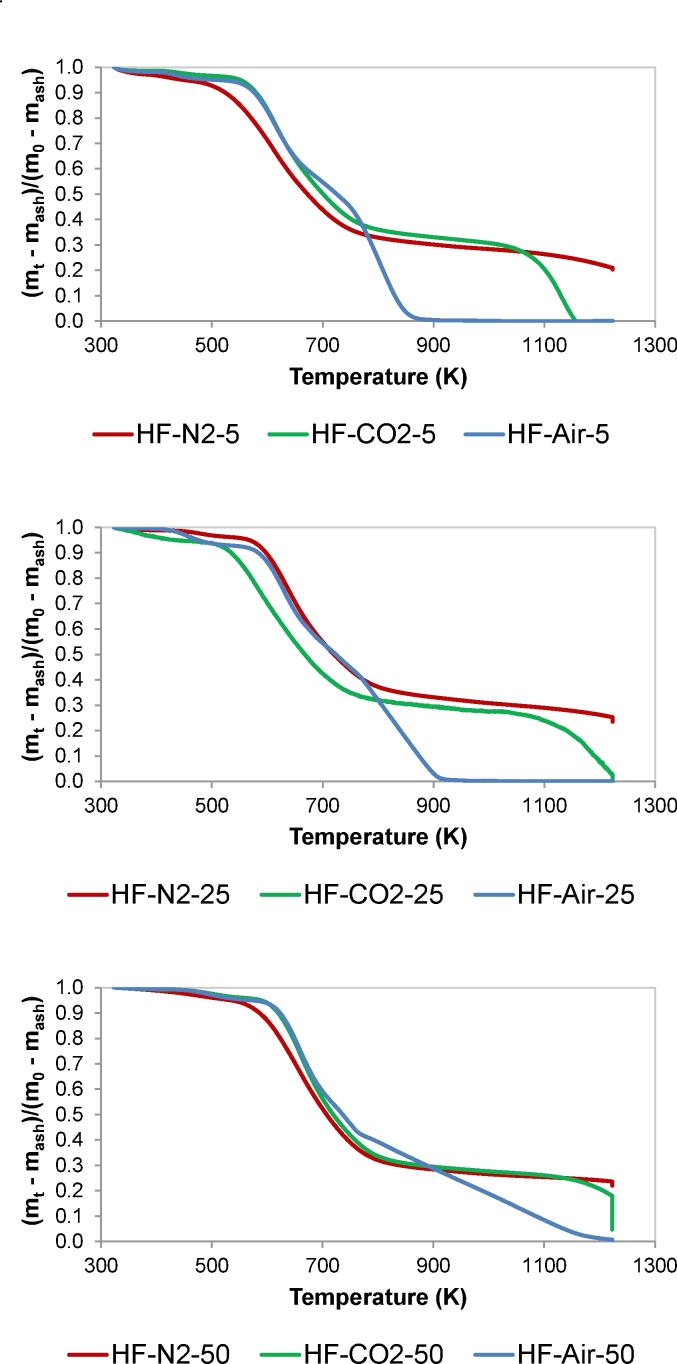
Effect of heating rate on the thermal behaviour (TG curves) of human faeces under pyrolysis (N_2_), gasification (CO_2_) and combustion (Air) conditions: (a) 5 K/min; (b) 25 K/min; and, (c) 50 K/min. Data are given in dry ash free basis.

Fig. 1 shows the influence of heating rate on the change of HF mass ratio (daf basis) with temperature under pyrolysis (N_2_), gasification (CO_2_) and combustion (Air) conditions. The three reaction atmospheres were tested because of the link between the three thermal processes. Pyrolysis constitutes the first stage of all solid fuel utilisation, and it is well known that pyrolysis conditions have an influence on the reactivity of the solid residue remaining after devolatilisation and, consequently, on gasification and combustion reactions [31,32].

Considering the case of 5 K/min, devolatilisation started around 500 K. Between 500 and 780 K, the mass loss rate was similar regardless the reaction atmosphere, which exhibits the prevalence of devolatilisation process within this temperature range. Under pyrolysis conditions and at temperatures higher than 780 K, the mass ratio slowly decreased up to complete release of the volatile matter. A similar behaviour was observed under CO_2_ atmosphere up to around 1000 K when gasification reaction occurred to complete conversion, which was achieved at approximately 1150 K. In the case of combustion of HF, the mass ratio decreased rapidly between 780 and 870 K, when the complete combustion of the burnable matter occurred. Similar behaviour was observed when evaluating the effect of the reaction atmosphere at 25 K/min and 50 K/min. Nevertheless, the higher the heating rate the higher the temperature needed for complete pyrolysis, gasification, and combustion. As explained by Kandiyoti et al. [31], when a fuel sample is heated rapidly, the speed of the temperature increase overtakes the sequence of the thermal events. Thus, slow heating of HF samples allowed completion of the thermal conversion stages, while rapid heating gave rise to step overlapping. Complete combustion was achieved at around 920 K and 1120 K when testing thermal conversion of HF at 25 K/min and 50 K/min, respectively.

The above results show that dedicated or retrofitted energy systems can be designed or optimised for thermal treatment of faecal sludge, provided such systems can manage faecal sludge in stages. Implications for the design of an appropriate energy conversion system includes: i) minimum temperatures of 475 K would be required for ignition to take place, ii) pre-treatment requirements such as drying must be kept below 475 K iii) slow thermal processing can enable complete conversion of HF, since it has a relatively slow decomposition rate. Considering that human faeces composition varies with nutritional intake, health, age, gender and mass index of individuals [4]; further tests can be carried out with variety of samples.

**Fig. 2:**
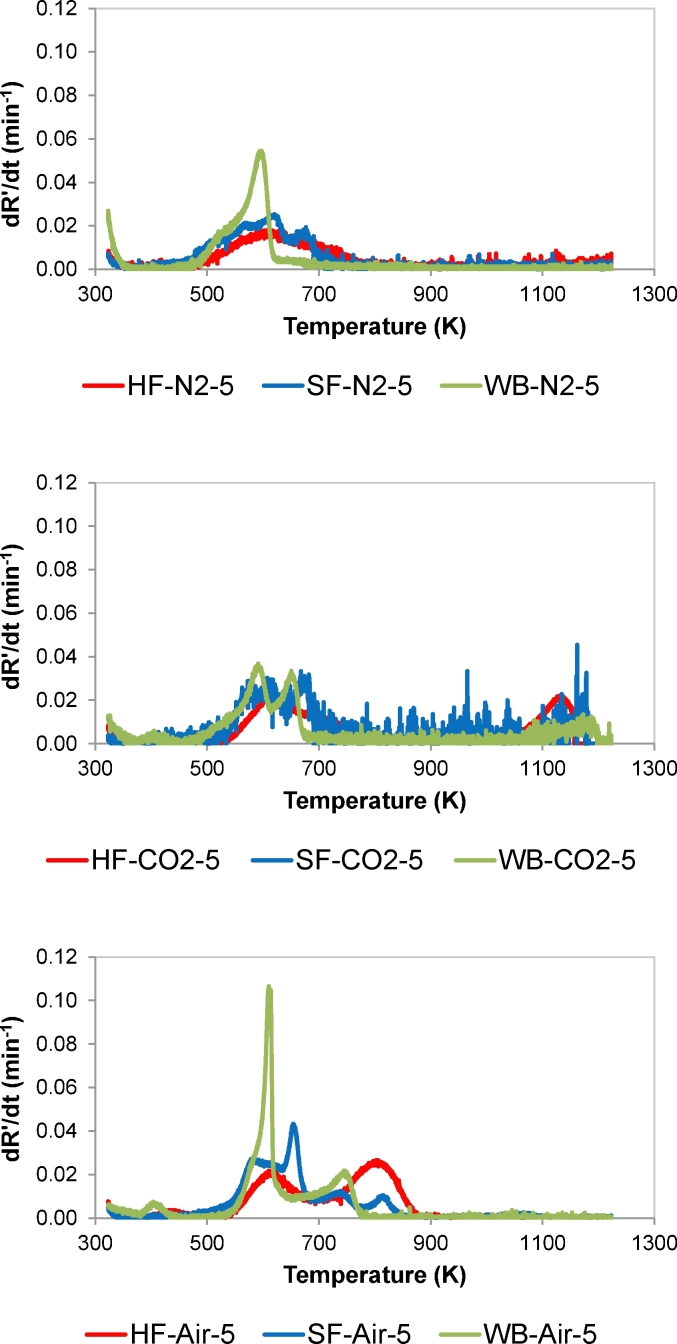
Effect of feedstock on the thermal behaviour (DTG curves) under: (a) pyrolysis (N_2_) conditions, (b) gasification (CO_2_) conditions, and (c) combustion (Air) conditions. HR = 5 K/min. Data are given in dry ash free basis.

The thermal behaviour of HF was compared to that of SF and WB by evaluating the differential thermogravimetric (DTG) curves shown in Fig. 2. Curves obtained at 5 K/min were used because this heating rate allowed for the completion of the thermal conversion stages. The pyrolysis DTG curves of HF and SF presented similar profiles, while the WB curve differed significantly. Pyrolysis of the three samples started around 475 K. In the case of WB, the maximum and complete release of volatiles occurred at around 595 K and 660 K respectively. The release of volatiles was slower for HF and SF, and completion occurred at around 790 K in the case of HF. These data confirm that pyrolysis of human faeces required a higher temperature to occur than pyrolysis of woody biomass.

In the case of the DTG curves obtained under CO_2_ atmosphere, only the HF sample exhibited a clear peak attributable to gasification reaction, with the maximum weight loss occurring at around 1125 K. In the case of WB, a peak evolved around 650 K. This peak was not observed under pyrolysis and combustion conditions and, consequently, could be related to the gasification process despite it evolved at a temperature rather low for gasification reaction to occur. Both WB and SF profiles presented a relatively flat peak with the maximum around 1175 K. These results point that the residue obtained from the devolatilisation of HF is more reactive under gasification conditions that from SF.

The assessment of the combustion curves showed an overlap of the pyrolysis and combustion events in the case of WB, which was not evident in the case of SF and did not occur in the case of HF. The combustion process of WB happened in two stages around 610 and 715 K respectively, and the complete burn out was observed at approximately 780 K. A higher temperature was needed for combustion to start in the case of HF. Thus, the peak around 610 K was mainly attributed to the release of volatiles while combustion process occurred between 710 and 880 K. In the case of SF, the weight loss associated to the combustion process occurred in three stages around 660, 740, and 820 K. Moreover, complete combustion of SF and HF occurred at around 850 and 880 K, respectively.

SF is said to have similar properties as typical HF, considering their physical and chemical properties [5]. This study has however showed that both SF and HF behave differently under thermal conditions, particularly under combustion. Material composition of SF includes yeast, propylene glycol, psyllium husk powder, calcium phosphate, cellulose powder etc [5,26]. As such, the multiple peaks in Fig. 2 can be attributed to progressive decomposition of these chemical compounds, particularly those with different thermal behaviour. A combined thermal and evolved gas analysis can identify these evolved chemical species, but this outside the scope of this work. Hence, the use of SF to address the combustion behaviour of HF may not provide accurate data. Conclusions drawn from experiments with SF should therefore be interpreted cautiously when applied to HF.

### 3.2 TGA results for human faeces blended with woody biomass

**Fig. 3:**
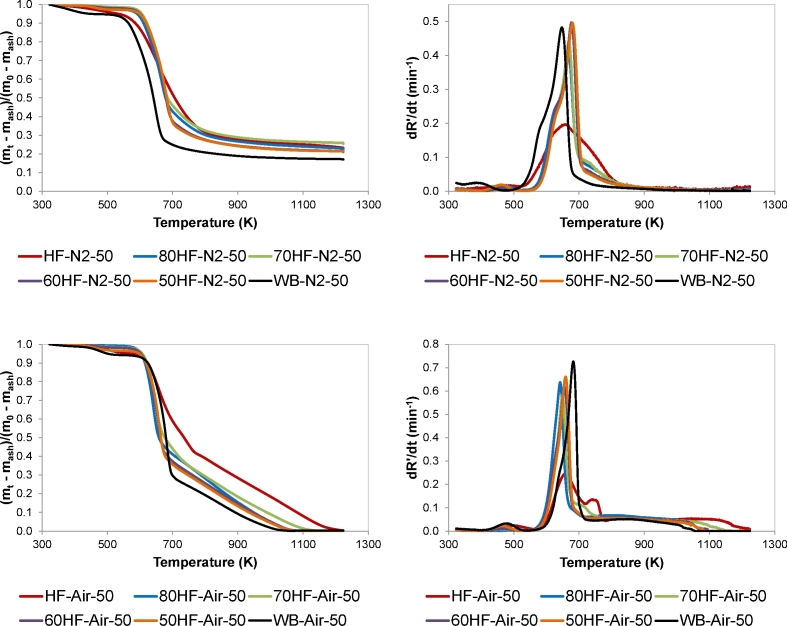
Effect of the content of HF on the thermal behaviour (TG and DTG curves) of HF and WB blends under: (a), (b) pyrolysis (N_2_) conditions, and (c), (d) combustion (Air) conditions. HR = 50 K/min. Data are given in dry ash free basis.

The change of mass ratio (TG and DTG curves) with temperature for the pyrolysis and combustion of various blends of HF and WB at a heating rate of 50 K/min is shown in Fig. 3. As can be seen, under N_2_ atmosphere, the TG curves of the HF and WB blends lay between those of the individual fuels at temperatures higher than 670 K. However, the blends required higher temperatures for the devolatilisation to start than those required by the individual HF and WB. TG combustion curves of the blends exhibited the same effect during the devolatilisation stage, and the curves only lay between those of the individual HF and WB for temperatures higher than 670 K. Liao and Ma [13] observed a similar effect when evaluating the co-combustion of coal and paper mill sludge, with the curves of the blends lying between those of the individual fuels for temperatures higher than 700 K. Nevertheless, the authors reported no explanation for this behaviour. On the contrary, other authors did not observe this effect when studying co-combustion of paper sludge and straw [12] or sewage sludge and coal [9,16], with the curves of the blends lying between those of the individual fuels for the whole range of temperatures.

As can be seen from the DTG curves, the contribution of WB to the thermal behaviour of the blends was clearer than the contribution of HF, even for blends with a content in faeces as high as 80 wt%. Thus, the DTG curves of the blends exhibited similar profiles to that of WB both under pyrolysis and combustion conditions. In the case of pyrolysis at 50 K/min, complete devolatilisation of HF required a broader range of temperature than WB (i.e. 500–850 K, and 500–750 K, respectively). The blends and the WB sample exhibited pyrolysis curves with similar shape, although the devolatilisation of the blends started at higher temperature, around 550 K, and was completed at approximately 850 K. In the case of combustion at 50 K/min, DTG curve of HF exhibited two peaks around 650 and 730 K, and WB curve presented a single peak at approximately 680 K. Similar to WB sample, the DTG curves of the blends presented a single peak. Otero et al. [10] also observed the thermogravimetric curves of the blends to be more similar to that of individual coal when assessing the co-combustion of coal and sewage sludge. Nevertheless, it is worth noting that these authors evaluated blends with a much lower content of sludge (up to 10 wt%) than the content of faeces (up to 80 wt%) used in this work. Although the blends exhibited a single peak similar to the WB sample, this was displaced towards lower temperatures as the content of HF increased. For example, 50HF-Air-50 presented the maximum at around 660 K while 80HF-Air-50 presented the maximum at 640 K, which points to an effect of the HF in reducing the temperature at which maximum combustion of the blends occurred. Liao and Ma [13] also observed that the combustion of paper mill sludge and coal blends became more similar to that of the sludge when the weight percentage of this component increased.

**Fig. 4:**
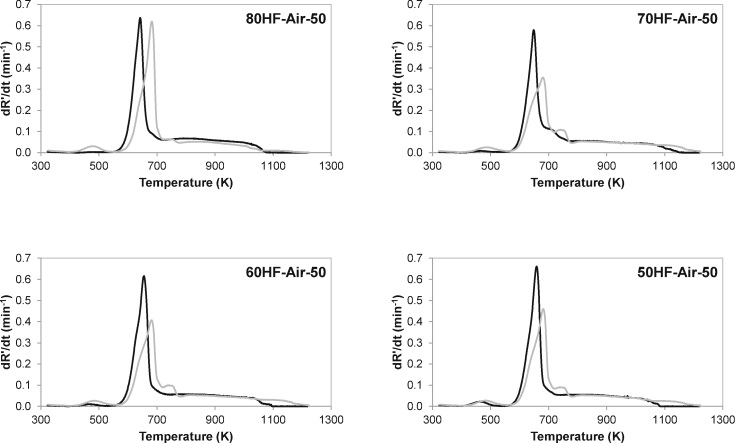
Comparison of experimental (black line) and calculated (grey line) thermal behaviour (DTG curves) of HF and WB blends under combustion (Air) conditions. HR = 50 K/min. Data are given in dry ash free basis.

The thermogravimetric behaviour of the blends of HF and WB points to interaction between the blends’ components. In order to confirm these interactions, DTG curves were calculated from the weighted sum of the blend components and compared to experimental DTG profiles (see Fig. 4). For all blends, the calculated DTG curves lagged behind the experimental curves. Xie and Ma [12] observed the same trend when comparing experimental and calculated TG curves from paper sludge and rice straw blends. The shift in the temperature of the main peak and the difference in the shape of the profile may be due to interactions between HF and WB in the blends during combustion. The main peak in the experimental DTG curves occurred at lower temperatures than that in the calculated blends and the intensity was higher than the expected by calculation. Moreover, the calculated DTG curves presented a secondary peak at higher temperature related to the HF contribution (see Fig. 3c), which was not observed in the actual experimental DTG curves. The discrepancy between the experimental and calculated curves are attributed to physical and chemical interactions between the blend components during thermal conversion, and it has been previously reported when evaluating the combustion of mixtures of sludge and other fuels [9,12,13,16].

### 3.3 Kinetic analysis

**Fig. 5:**
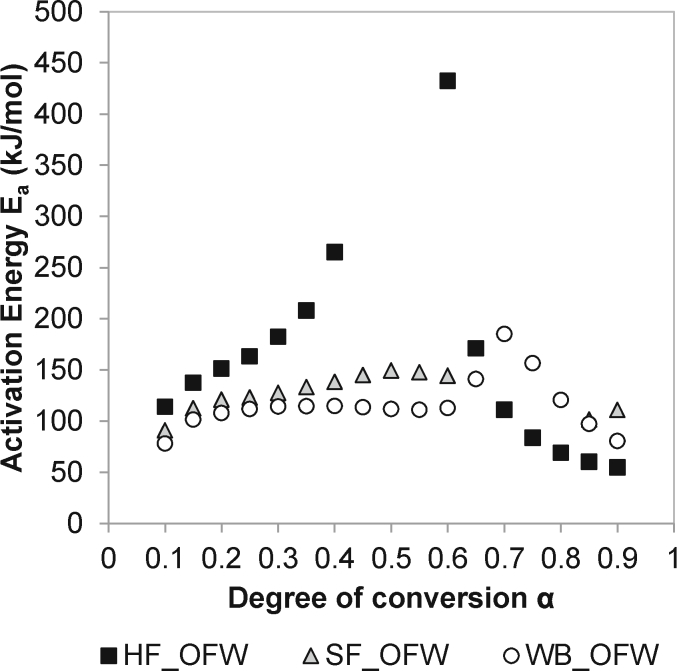
Activation energy, E_a_, distribution at different conversion rates, α, determined from the FWO method for the combustion of human faeces (HF), simulant faeces (SF) and woody biomass (WB) samples.

Fig. 5 shows the activation energy values, E_a_, versus the degree of conversion, α, for the combustion of HF, SF and WB calculated by means of the OFW method. The activation energy was calculated through corresponding temperature at each fixed degree of conversion and heating rate. Degrees of conversion ranging from 10% to 90% were considered in this work. Fig. 5 reveals the multi-step kinetics of the process, which reflect the complex mechanisms of pyrolysis and combustion and the associated changes in composition of the fuel during the process [12,13,20]. The three samples exhibited a maximum E_a_ value, although it was more than 250 kJ/mol higher in the case of HF sample compared to SF and WB samples. In addition, the maximum value was obtained at different degrees of conversion, i.e. α = 0.6 in the case of HF (corresponding to temperatures between 765 and 790 K) and α = 0.7 in the case of WB (corresponding to temperatures between 660 and 700 K). Chen et al. [20] also observed a dramatic increase in the energy of activation with the degree of conversion when investigating the pyrolysis of cattle manure, and found a maximum OFW activation energy value of 445 kJ/mol at α = 0.75. The authors related the increment of E_a_ value with the occurrence of more difficult conversion processes as the degree of conversion increases.

The average activation energy values were also calculated by means of the Vyazovkin method for comparison purposes. Similar average activation energy values were obtained from OFW and Vy methods, although the latter gave slightly higher E_a_ values for all samples (see Table 1). The average activation energy of HF was approximately 35% higher than that corresponding to WB, which agrees with the higher temperatures required for the complete combustion of HF. The average activation energy of HF obtained in this work was higher than OFW values reported by Sanchez et al. [23] for sewage sludge (146.4 kJ/mol) and animal manure (143.3 kJ/mol). Moreover, the average activation energy of HF was approximately 20% higher than that corresponding to SF, which reinforces the idea that the thermal behaviour of simulant faeces does not necessarily resemble that of human faeces. In fact, the average activation energy of SF was in the line with OFW activation energy values reported Otero et al. [10] for two different samples of sewage sludge (i.e. 99.3 and 138.1 kJ/mol). The higher average activation energy of HF can be attributed to the highly bound nature of the material. HF is a by-product of digestion and a blend of undigested food materials, microbial biomass, digestive juices and secretions, along with soluble and insoluble nutrients including fats, protein, water and polysaccharides [33]. These materials have gone through complex processes —evolving from ingestion, mechanical and chemical digestion, absorption to compaction of the waste. SF on the other hand is a combination of food-grade substances that are loosely bound together. As such, the energy requirement for breaking the chemical compounds in HF can be expected to be much higher than SF. Further investigations can be carried to examine how different faeces (watery to hard type stools) behave under thermal conditions, and how the activation energy of these materials changes.

**Table 1:** Average activation energy values obtained by the OFW and Vy methods for the combustion human faeces (HF), simulant faeces (SF) and woody biomass (WB) samples.

Sample	E_a_ (kJ/mol) - OFW	E_a_ (kJ/mol) - Vy
HF	157.4	159.3
SF	126.6	128.5
WB	116.1	116.7

Table 2 summarizes the reaction order *n* calculated by means of Avrami's equation as a function of temperature for HF, SF and WB. In general, the reaction order value decreased with temperature to a minimum and then increased. Nevertheless, the variation of *n* value was different for each sample. The minimum was achieved at 773 K in the case of HF, at 973 K in the case of SF and at 1073 K for WB. The values ranged from very close to zero (pseudo zero-order reaction) at the minimum to 0.6 for HF, 0.5 for SF and 0.9 for WB. Similar to the E_a_ values, the dependence of the *n* values with the degree of conversion pointed that the process consisted of multiple steps. Moreover, non-integer orders were obtained at all temperatures, which indicates the complex reaction mechanism of the thermochemical conversion processes of these materials. The WB sample showed higher average reaction order value than those for HF and SF. Sanchez et al. [23] found average *n* values of 0.25 for animal manure and 0.17 sewage sludge between 473 and 673 K. These values are close to the average value achieved for HF within the same temperatures, i.e. *n* = 0.2, while the value for SF in the same temperature range was higher, i.e. *n* = 0.4.

**Table 2:** Reaction order obtained by the Avrami's method at different temperatures for the combustion human faeces (HF), simulant faeces (SF) and woody biomass (WB) samples.

Temperature (K)	HF	SF	WB
373	0.6	0.4	0.9
473	0.4	0.4	0.9
573	0.2	0.5	0.6
673	0.1	0.3	0.3
773	0.05	0.1	0.6
873	0.6	0.2	0.6
973	0.5	0.07	0.3
1073	0.6	0.1	0.09
Average *n* (473–673 K)	0.2 ± 0.1	0.4 ± 0.1	0.6 ± 0.3
Average *n* (373–1073 K)	0.4 ± 0.2	0.3 ± 0.2	0.6 ± 0.3

## 4 Conclusions

In this work, experimental investigation of the thermochemical conversion of human faeces was carried out by means of thermogravimetric analyses. Thermal conversion was carried out under N2, CO2, and air atmospheres to model pyrolysis, gasification, and combustion conditions, and at three different heating rates, i.e. 5, 25 and 50 K/min. Similar analyses were performed on simulant faeces, woody biomass, and blends of human faeces and woody biomass for comparison purposes.

The TG and DTG curves showed that the devolatilisation of HF requires higher temperatures and relatively slow to those of WB. HF and SF showed similar behaviour under pyrolysis conditions. However, the residue obtained from pyrolysis of HF was found to be more reactive than that from SF under CO2 gasification conditions. SF exhibited different combustion characteristics to HF and WB. While the combustion of WB and HF happened in two stages, about (610 and 715 K) and (710 and 880 K) respectively, the combustion of SF occurred in three stages, around 660, 740, and 820 K. These results imply that conclusions drawn from experiments with SF should be interpreted cautiously when applied to HF. The co-processing of HF and WB showed that these materials interact during thermal processes, with more evident contribution of WB to the thermal behaviour of the blends than that of HF. The increasing content of HF in the blend decreased the maximum combustion temperature.

The activation energy for HF, WB and SS were determined using the OFW and Vyazovkin non-isothermal model-free kinetic methods. Avrami's equation was used to study the reaction order. The average activation energy was higher for HF (Ea = 157.4 kJ/mol) than for SF (Ea = 126.6 kJ/mol) and WB (Ea = 116.1 kJ/mol). Across the tested temperature range, the average reaction was similar for HF (n = 0.4) and SF (n = 0.3), but lower than that of WB (n = 0.6). This paper provides new kinetic data on the thermochemical conversion of human faeces and is relevant for the design of an appropriate energy system for thermal treatment of faecal sludge and related materials.
